# Facile preparation of Au- and BODIPY-grafted lipid nanoparticles for synergized photothermal therapy

**DOI:** 10.3762/bjnano.13.118

**Published:** 2022-12-02

**Authors:** Yuran Wang, Xudong Li, Haijun Chen, Yu Gao

**Affiliations:** 1 Cancer Metastasis Alert and Prevention Centre, College of Chemistry and Fujian Provincial Key Laboratory of Cancer Metastasis Chemoprevention and Chemotherapy, Fuzhou University, Fuzhou 350108, Fujian, China; 2 College of Chemistry, Key Laboratory of Molecule Synthesis and Function Discovery (Fujian Province University), Fuzhou University, Fuzhou 350116, Fujian, China

**Keywords:** BODIPY, gold nanoparticles, lipid nanoparticles, photothermal therapy, synergism

## Abstract

Gold nanoparticles with large size exhibit preferable properties for photothermal therapy (PTT). However, the prolonged tissue retention and slow elimination of gold nanoparticles limit their therapeutic applications. Previously, gold nanoclusters carrying lipid nanoparticles (Au-LNPs) have been reported after simply mixing Au^3+^ with preformed diethylenetriaminepentaacetic acid lipid nanoparticles to solve this contradiction. Au-LNPs demonstrated enhanced photothermal effects in comparison to neat gold nanoparticles. To further improve the photothermal activity, we introduced the organic photothermal agent boron dipyrromethene (BODIPY) to Au-LNPs for synergistic PTT. Au- and BODIPY-grafted LNPs (AB-LNPs) were formed by simply mixing Au-LNPs with BODIPY. The BODIPY could be associated stably to Au-LNPs, and the release of BODIPY from AB-LNPs could be accelerated by laser irradiation. AB-LNPs are scalable and showed excellent photothermal effects. AB-LNPs showed enhanced cellular uptake efficiency compared to free BODIPY in 4T1 breast cancer cells. Under laser irradiation, AB-LNPs exhibited synergistic photothermal effects with significantly reduced dosage compared to monotherapy (treatments with Au-LNPs or free BODIPY alone). This study thus provides a facile and adaptive strategy for the development of a scalable and safe high-performance nanoplatform for synergistic PTT in the treatment of cancer and other diseases.

## Introduction

Photothermal therapy (PTT) relies on photothermal agents (PTAs) to convert light into heat energy to burn cancer cells. Due to its spatial specificity and minimal invasiveness, it has attracted a great deal of attention as complementary modality for conventional cancer therapy options [1]. Gold nanoparticles (AuNPs) can absorb light and generate heat from light absorption because of the surface plasmon resonance (SPR) phenomenon and the tunable near-infrared (NIR) absorption [2]. Various gold nanoscale platforms, including nanostars, nanorods, nanospheres, nanoshells, and nanocages, have been designed as PTAs for PTT. Gold nanorods (ca. 50 nm), nanoshells (ca. 130 nm), and nanoparticles with large sizes (>50 nm) are suitable for PTT because of strong NIR absorption [3]. However, particles with larger size are likely to have long resident times and slow elimination in tissues, which may lead to long-term toxicity. Commercially available AuNPs with particle sizes of 10–30 nm can be synthesized by the classical Turkevich method [4]. Rats that received a single intravenous dose of commercial AuNPs still showed high gold concentrations in the liver at day 28 [5].

To reduce tissue retention of AuNPs, ultrasmall gold nanoclusters with renal clearance ability were developed for imaging and sensing [6–7]. Nevertheless, they were not reported for PTT use. In a previous reported work, we synthesized tiny gold nanoclusters by simply mixing Au^3+^ with preformed lipid nanoparticles (LNPs) containing diethylenetriaminepentaacetic acid (DTPA) [8]. The Au-grafted LNPs (Au-LNPs) showed significantly enhanced photothermal effects in comparison to the commercial AuNPs. The numerous ultrasmall gold nanoclusters stably anchored on the LNPs yield a concentrated heat generation of Au-LNPs under NIR irradiation. However, Au-LNPs are still not ideal for PTT because of the limited temperature elevation (only to ca. 25 °C). The anti-proliferative activities of Au-LNPs under NIR irradiation were enhanced when gamma irradiation was administered additionally.

In order to achieve better PTT effects, the combined use of different PTAs could yield synergistic photothermal effects [9–10]. PTAs can be generally divided into inorganic and organic PTAs. As organic PTAs, boron dipyrromethenes (BODIPYs) have attracted increasing attention because of high molar absorption coefficient and photochemical stability [11]. Besides, fabricating BODIPYs with halogenated substituents can improve the photothermal conversion efficiency during PTT [12]. However, BODIPYs are hydrophobic PTAs with rigid structure and poor water solubility [11–12]. Therefore, several lipid nanoparticulate drug delivery systems have been reported to encapsulate BODIPYs to improve their water solubility and photostability while retaining their original photothermal effects [13–14]. BODIPY-AuNP conjugates and BODIPY-AuNP composites were also reported for diagnosis and photodynamic therapy (PDT) [15–17].

**Scheme 1:**
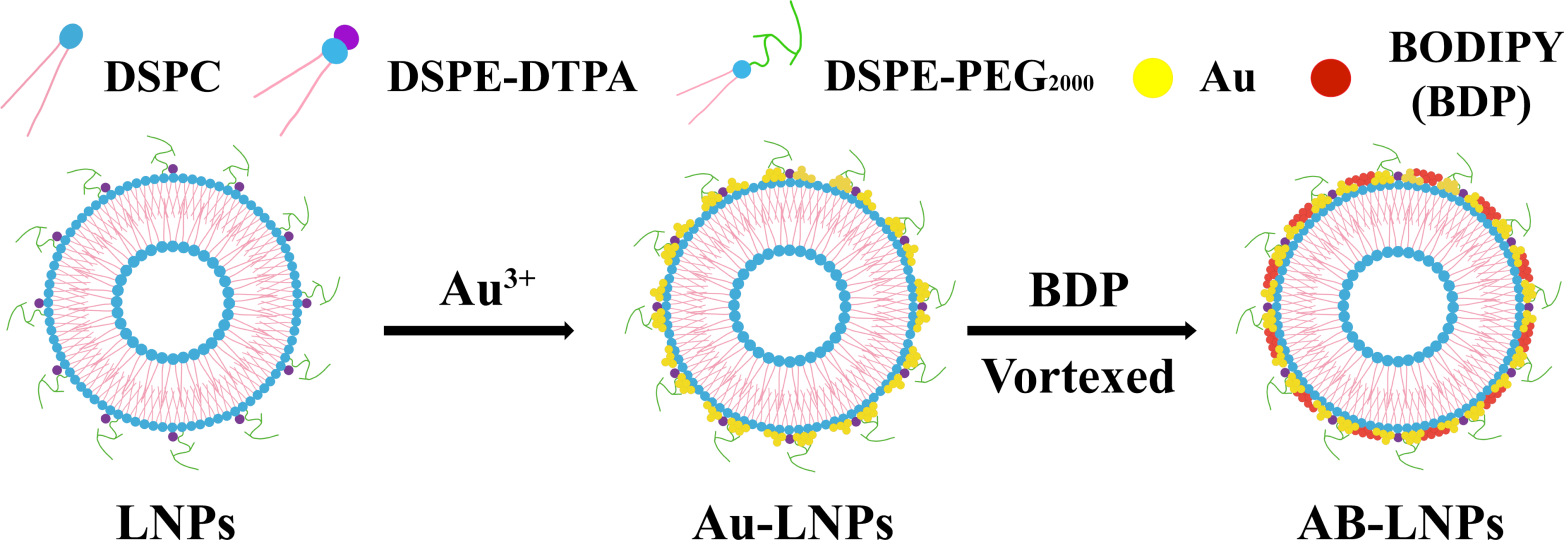
Schematic diagram of the preparation of AB-LNPs.

Hence, we aimed to synthesize a common halogenated BODIPY compound (BDP) and further modified Au-LNPs with BDP for synergized PTT (Scheme 1). According to [8], we prepared Au-LNPs with optimized particle size and polydispersity index (PDI), and then mixed BDP with Au-LNPs to obtain both Au- and BDP-grafted LNPs (AB-LNPs). The physiochemical characteristics, the photothermal properties, and the stabilities of AB-LNPs were studied in detail. The cellular uptake efficiency and the synergized photothermal effects of this new nanosystem were also evaluated in the following studies.

## Materials and Methods

### Materials

1,2-Distearoyl-*sn*-glycero-3-phosphocholin (DSPC) and 1,2-distearoyl-*sn*-glycero-3-phosphoethanolamine-*N*-DTPA (DSPE-DTPA) were purchased from Avant Polar Lipids (Alabaster, AL, USA). 1,2-Distearoyl-*sn*-glycero-3-phosphoethanolamine-*N*-[poly(ethylene glycol)2000] (DSPE-PEG_2000_) was obtained from Genzyme Pharmaceuticals (Cambridge, MA, USA). 3-(4,5-Dimethylthiazol-2-yl)-2,5-diphenyltetrazolium bromide (MTT) and Hoechst 33342 were obtained from Sigma-Aldrich. Gold(III) chloride trihydrate (HAuCl_4_·3H_2_O) was purchased from Sinopharm Chemical Reagent (China). Fetal bovine serum (FBS) was obtained from Zhongke Xinchuang Biotechnology (Fuzhou, China). RPMI-1640 medium was purchased from Meilun Biotechnology (Dalian, China). Trifluoroacetic acid (TFA), 2,3-dichloro-5,6-dicyano-1,4-benzoquinone (DDQ), dichloromethane (DCM), dimethyl sulfoxide (DMSO), triethylamine (TEA), *N*-iodosuccinimide (NIS), acetonitrile (CH_3_CN), 4-methoxybenzaldehyde, piperidine, petroleum ether (PE), and ethyl acetate (EA) were provided by Sinopharm Chemical Reagent (China). Boron trifluoride diethyl etherate (BF_3_·OEt_2_) and 2,4-dimethylpyrrole were freshly purified by distillation under reduced pressure.

### Synthesis and characterization of BDP

All chemical reagents were obtained from commercial suppliers and used without further purification. ^1^H nuclear magnetic resonance (NMR) and ^13^C NMR spectra were measured on a Bruker AVANCE 400 spectrometer (400 MHz).

**Scheme 2:**
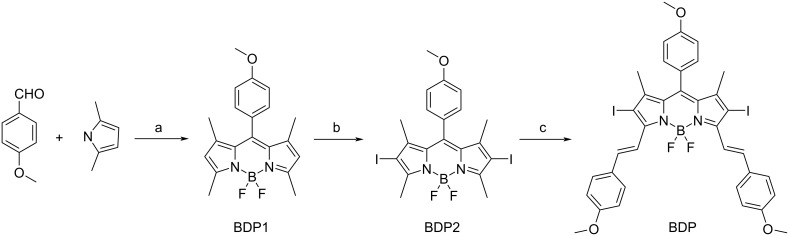
Synthesis of BDP. (a) 2,4-dimethylpyrrole, TFA, DCM, 25°C, 8 h; DDQ, DCM, 25 °C, 0.5 h; TEA, BF_3_·OEt_2_, 25 °C, 2 h; (b) NIS, DCM, 25 °C, 1 h; (c) 4-methoxybenzaldehyde, piperidine, CH_3_CN, 80 °C, 2 h.

The synthesis of BDP is shown in Scheme 2. BDP1 was synthesized in accordance with a previous report [18]. ^1^H NMR (500 MHz, CDCl_3_) δ 7.15 (d, *J* = 5.4 Hz, 2H), 6.99 (d, *J* = 6.5 Hz, 2H), 5.96 (s, 2H), 3.86 (s, 3H), 2.54 (s, 6H), 1.42 (s, 6H); ^13^C NMR (150 MHz, DMSO-*d*_6_) δ 160.3, 155.1, 143.2, 142.6, 131.6, 129.6, 126.4, 121.7, 115.1, 55.7, 14.7; HRESIMS *m*/*z*: [M + H]^+^ calcd, 355.1788; found, 355.1791.

BDP2 was prepared as reported in a previously reported work [19]. ^1^H NMR (400 MHz, CDCl_3_) δ 7.16 (d, *J* = 8.6 Hz, 2H), 7.06 (d, *J* = 8.6 Hz, 2H), 3.92 (s, 3H), 2.66 (s, 6H), 1.47 (s, 6H); ^13^C NMR (125 MHz, CDCl_3_) δ 160.6, 153.7, 142.3, 140.6, 130.9, 130.8, 129.1, 128.8, 126.3, 114.8, 111.7, 55.4, 13.8, 13.6; HRESIMS *m*/*z*: [M + H]^+^ calcd, 606.9721; found, 606.9729.

BDP was synthesized in accordance with a previous report with minor modifications [20]. To a solution of BDP2 (0.15 mmol, 90.9 mg) in CH_3_CN (5 mL) was added 4-methoxybenzaldehyde (0.9 mmol, 122.4 mg) and 0.2 mL of piperidine. The reaction was stirred at 80 °C for 2 h. Then the resulting mixture was poured into saturated NaHCO_3_ (20 mL) and extracted with DCM (40 mL) for three times, dried over anhydrous Na_2_SO_4_, filtered, and concentrated under reduced pressure to afford the crude product. The crude product was purified via silica gel column chromatography using DCM/PE = 1:1 as eluent to obtain a green solid. ^1^H NMR (500 MHz, CDCl_3_) δ 8.06 (d, *J* = 16.5 Hz, 2H), 7.54 (d, *J* = 8.7 Hz, 4H), 7.49 (s, 2H), 7.11 (d, *J* = 8.6 Hz, 2H), 6.99 (d, *J* = 6.9 Hz, 2H), 6.89 (d, *J* = 8.7 Hz, 4H), 3.84 (s, 3H), 3.79 (d, *J* = 2.9 Hz, 6H), 1.43 (d, *J* = 3.8 Hz, 6H); HRESIMS *m*/*z*: [M + H]^+^ calcd, 843.0558; found, 843.0515.

### Preparation and characterization of AB-LNPs

LNPs containing DTPA with negative tetravalency were prepared using a previously reported method [21]. LNPs were prepared with a molar ratio of DSPC/DSPE-PEG/DSPE-DTPA = 18:2:5. Au-LNPs were synthesized using our previously reported method [8]. To prepare Au-LNPs, the LNPs in suspension were mixed with Au^3+^ for at least 6 h at room temperature. BDP was first dissolved in DMSO in a centrifuge tube to a concentration of 3.38 mM. The liquor was diluted 20 times with water and then sonicated for 10 min. The AB-LNPs was prepared by simply physical complexation. The diluted BDP aqueous suspension was then added drop by drop into the Au-LNPs aqueous suspension. The mixture was vortexed for 30 s and further sonicated for 10 min to obtain AB-LNPs.

### Characterization of AB-LNPs

The size distributions and zeta potentials were measured using a dynamic light scattering (DLS) technique on Malvern Zetasizer (Malvern Instruments Ltd., UK). Ultraviolet–visible (UV–vis) absorption spectra were measured with an UV–vis spectrometer (Q-5000, Quawell, America). The amounts of BDP in AB-LNPs were analyzed using UV–vis spectroscopy after the dissolution of AB-LNPs in DMSO by measuring the absorbance at a wavelength of 600 nm. The loading efficiency (LE, %) was determined and calculated using the following formula:


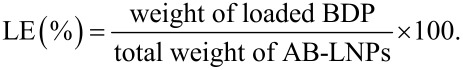


The morphology of AB-LNPs was obtained on a Hitachi HT7700 transmission electron microscope (TEM, Hitachi, Japan). AB-LNPs were diluted and plated on a carbon-coated copper grid.

### The stability of AB-LNPs

The stability of AB-LNPs was studied by measuring mean particle size and zeta potential of the samples in phosphate-buffered saline (PBS) or in RPMI-1640 medium with 10% FBS over a period of 7 days. Samples were collected at specific time points to monitor the changes in particle size and zeta potential.

### In vitro drug release properties of AB-LNPs

The release properties were evaluated in PBS with different pH values (pH 7.4 and 5.5). AB-LNPs dispersion (2 mL) in a dialysis bag was placed in 20 mL release medium. After 2 h and after 6 h, the sample was irradiated with a 680 nm laser (0.5 W/cm^2^, 60 s). At predetermined time points, 1 mL of solution was taken out to measure the amount of the released drug by UV–vis spectroscopy, and an equal volume of fresh release medium was added to keep the volume constant.

### Photothermal measurement for AB-LNPs

To study the photothermal properties of AB-LNPs, different concentrations of AB-LNPs with BDP concentrations of 25, 50, and 100 μM were irradiated with laser irradiation (680 nm, 0.5 W/cm^2^) for different time intervals. The temperature changes of AB-LNPs with BDP concentration of 100 μM were also measured under laser irradiation with different power densities (0.25, 0.4, and 0.5 W/cm^2^). The temperature changes of water, BDP, Au-LNPs, and AB-LNPs with a BDP concentration of 100 μM and a Au concentration of 100 μM with laser irradiation (680 nm, 0.5 W/cm^2^) were also recorded for 10 min. The temperature of the samples for photothermal conversion measurements was recorded by an infrared thermal camera (FOTRIC 225 s, USA).

### Cell culture

Murine breast cancer 4T1 cell line was purchased from Cell Resource Center of Shanghai Institute for Biological Sciences (Chinese Academy of Sciences, Shanghai, China). 4T1 cells were cultured in RPMI-1640 medium containing 100 μg/mL streptomycin, 100 U/mL penicillin, and 10% FBS. The cells were cultured in a humidified incubator with atmosphere of 5% CO_2_ at 37 °C.

### Cellular uptake studies

4T1 cells were grown in 12-well plates and cultured for 24 h. The cells were incubated with free BDP (30 μM) and AB-LNPs (with a BDP concentration of 30 μM and a Au concentration of 30 μM) at 37 °C for 0.5, 1, 2, 4, 6, and 8 h. Then, the cells were washed, digested, and suspended in 500 μL PBS. The fluorescence was detected by flow cytometry (Becton Dickinson FACSAriaIII cell sorter) in PerCP-Cy5.5 channel to detect the ﬂuorescence of BDP according to our previous report [22].

4T1 cells were seeded onto 12-well plates with a cover glass placed on the bottom of each well. After culture for 24 h, cells were incubated with free BDP (30 μM) and AB-LNPs (with a BDP concentration of 30 μM and a Au concentration of 30 μM) for 6 h. After washing twice with PBS, the cells were incubated with Hoechst 33342 (10 µg/mL) for 15 min and fixed with 4.0% (w/v) paraformaldehyde for 15 min at room temperature in the dark. Finally, the cells were washed twice with PBS and visualized under a confocal laser scanning microscope (CLSM, Leica TCS SP8, Germany).

### Cytotoxicity and synergized photothermal effects

4T1 cells were seeded onto 96-well plates at a density of 1.0 × 10^4^ cells per well and cultured for 24 h. Various concentrations of AB-LNPs were added into each well. After being cultured for 6 h, the irradiation groups were exposed to 680 nm laser irradiation (0.5 W/cm^2^) for 60 s. The plates were further incubated at 37 °C for 24 h. The cell viability was then measured by MTT assay according to our previous report [23].

### Statistical analysis

All data shown in this article are expressed as mean ± SD for at least three separate experiments. Statistical analysis was performed using the Student’s *t*-test or one-way analysis of variance (ANOVA). The differences were considered signiﬁcant for *p* < 0.05, and *p* < 0.01 was indicative of a very signiﬁcant difference.

## Results

### Optimizing the reaction order to prepare AB-LNPs

**Scheme 3:**
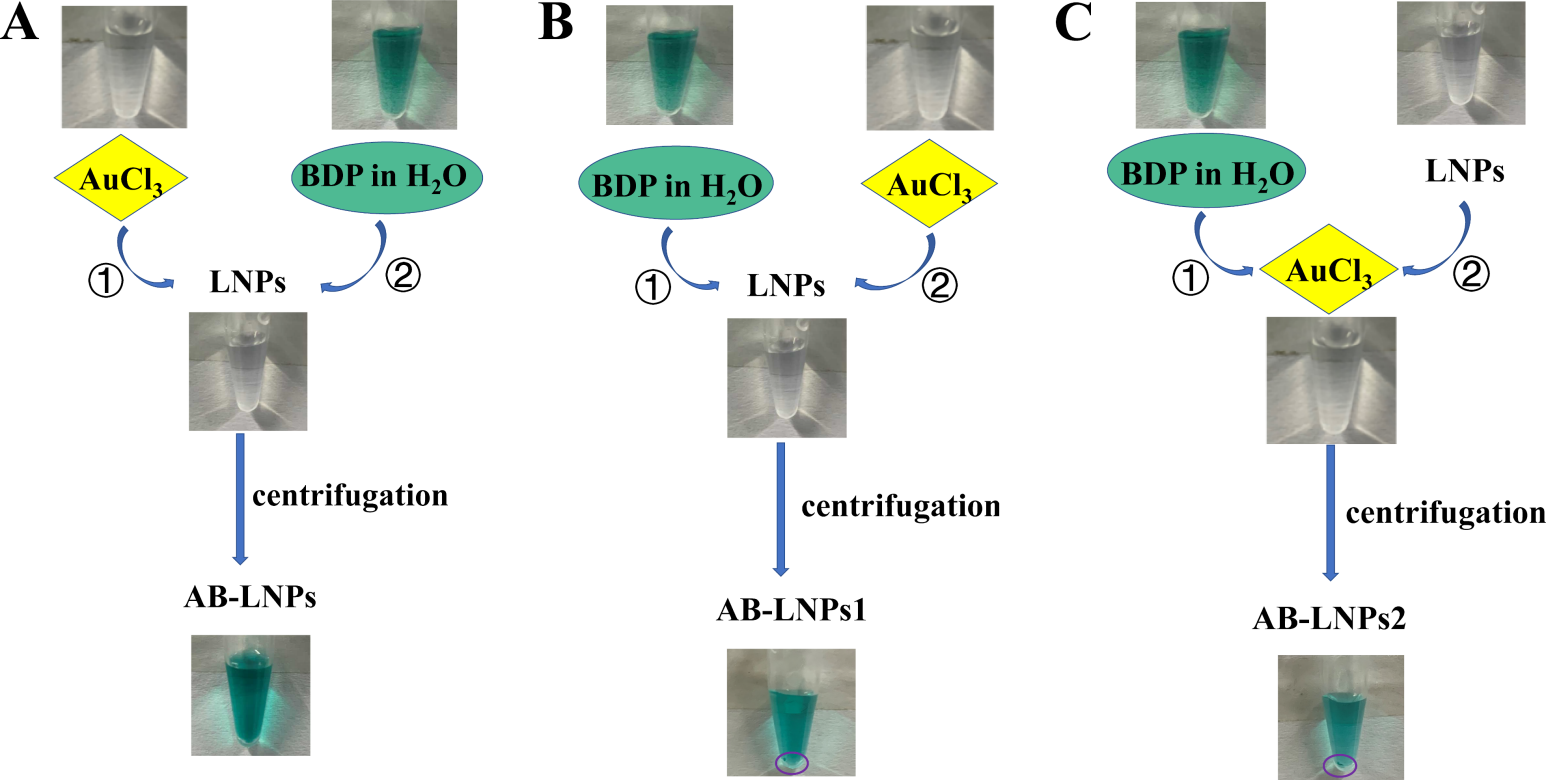
Preparation orders and appearance of different formed AB-LNPs. Method A: Au^3+^ was added to the preformed LNPs to form Au-LNPs followed by addition of BDP water suspension. Method B: BDP was added to the preformed LNPs to form B-LNPs followed by addition of Au^3+^. Method C: Au^3+^ was mixed with BDP, then the mixture was added to the preformed LNPs. LNPs were prepared with a molar ratio of DSPC/DSPE-PEG/DSPE-DTPA = 18:2:5. The molar ratio of DSPE-DTPA/Au^3+^/BDP in all preparations was 2:1:1. AB-LNPs1 and AB-LNPs2 readily aggregate. AB-LNPs demonstrates good suspension stability.

Drug molecules can self-assemble into nanoparticles or be loaded into drug delivery systems through van der Waals forces, hydrogen bonds, π–π stacking, or electrostatic or hydrophobic interactions [24]. Several BODIPYs have been reported to be loaded into liposomes for cancer therapy [25]. Therefore, we speculated that BODIPY can be associated with our previously reported preformed LNPs. We chose a common halogenated BODIPY compound, BDP, to work with Au-LNPs to obtain both Au- and BDP-grafted LNPs for synergistic PTT. We first evaluated the role of the preparation order on the formation of AB-LNPs. Three different orders used are schematically presented in Scheme 3. The first reaction order (method A) is mixing Au^3+^ with preformed LNPs to form Au-LNPs (the synthesis procedures for Au-LNPs were reported previously [8]) followed by addition of an aqueous BDP suspension. The blue dispersion of Au-LNPs turned green after the addition of BDP. After mixing Au-LNPs with BDP (molar ratio of DSPE-DTPA/Au^3+^/BDP = 2:1:1), the mixture was vortexed for 30 s and further ultrasonicated for 10 min at 25 °C. The acquired green dispersion, designated as AB-LNPs, showed excellent stability with no precipitation after centrifugation. Method B is mixing BDP with preformed LNPs followed by addition of Au^3+^. After the same vortex and ultrasonication, the obtained product, designated as AB-LNPs1, exhibited green color in suspension. However, green precipitates could be collected after centrifugation, suggesting the dissociation of BDP or Au from LNPs. Green precipitates also could be found in the products (designated as Au-LNPs2) prepared using a third method (method C) by addition of the mixture of Au^3+^ and BDP into preformed LNPs. To sum up, AB-LNPs prepared using method A showed good stability compared with Au-LNPs1 and Au-LNPs2. The preparation method is simple and could be scalable. Therefore, we chose method A to prepare AB-LNPs for further studies.

### Optimizing composition to prepare AB-LNPs

**Figure 1:**
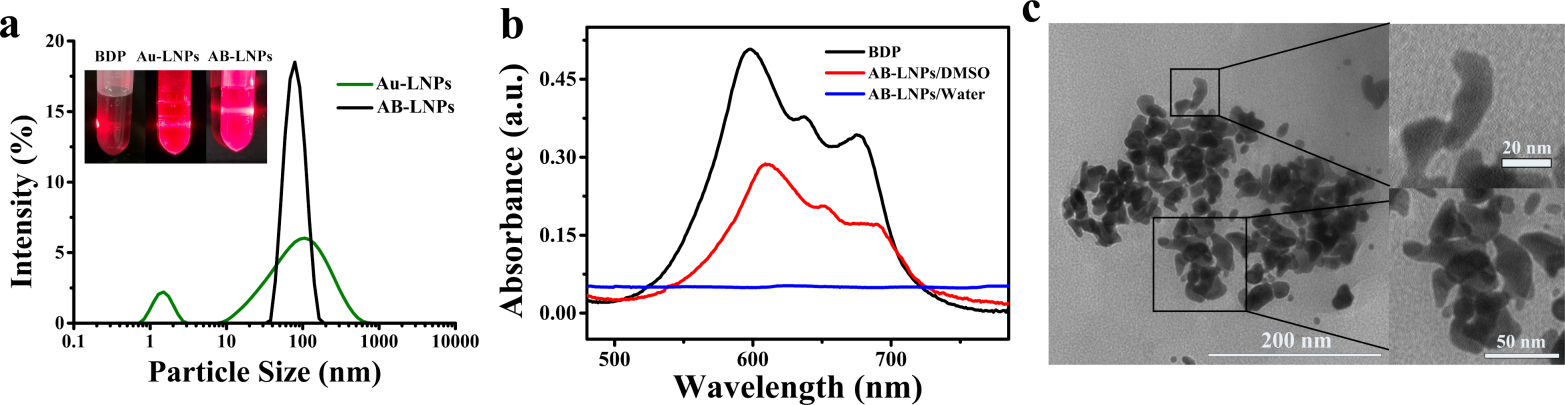
The characterization of AB-LNPs. (a) Size distribution of Au-LNPs and AB-LNPs. Digital photo of AB-LNPs showing distinct Tyndall effects. (b) UV–vis spectra of BDP and AB-LNPs. (c) TEM image of AB-LNPs.

The properties of Au-LNPs were closely related to the DSPE-DTPA/Au^3+^ molar ratio [8]. Mean particle size and PDI of Au-LNPs varied with the change of the DSPE-DTPA/Au^3+^ molar ratio. No peak corresponding to large particles (>100 nm) was observed when the DSPE-DTPA/Au^3+^ molar ratios were 1:1 and 2:1. The particle sizes of the Au-LNPs were approximately 50–100 nm, and the PDI was smaller than 0.3. We, hence, chose Au-LNPs samples prepared under these two molar ratios for the subsequent composition optimizing. The particle size and PDI of AB-LNPs were closely related to the DSPE-DTPA/BDP molar ratio (Table 1). With the increase of the DSPE-DTPA/BDP molar ratio, the particle sizes and PDIs of AB-LNPs decreased first and then increased. The optimal DSPE-DTPA/Au^3+^/BDP molar ratio is 2:1:1. The particle size and PDI of AB-LNPs under the optimal DSPE-DTPA/Au^3+^/BDP molar ratio of 2:1:1 are 72.87 ± 1.51 nm and 0.150 ± 0.028, respectively. Compared to Au-LNPs with two populations, AB-LNPs showed only one normal distribution peak (Figure 1a), demonstrating that the addition of BDP could increase the monodispersity of Au-LNPs. Au-LNPs and AB-LNPs exhibited the Tyndall light-scattering effect, but free BDP did not show the Tyndall effect (Figure 1a), which further proving the successful formation of AB-LNPs.

**Table 1:** DLS size analysis of AB-LNPs. Data are shown as mean ± SD (*n* = 3).

DSPE-DTPA/Au^3+^ molar ratio	DSPE-DTPA/Au^3+^/BDP molar ratio	Particle size (nm)	Polydispersity index (PDI)

1:1	1:1:0.2	128.22 ± 2.72	0.416 ± 0.018
	1:1:0.5	84.62 ± 0.62	0.221 ± 0.035
	1:1:1	83.22 ± 0.42	0.206 ± 0.016
	1:1:2	113.01 ± 1.06	0.343 ± 0.026
	1:1:5	130.11 ± 1.03	0.333 ± 0.020

2:1	2:1:0.2	102.54 ± 1.50	0.187 ± 0.018
	2:1:0.5	81.27 ± 1.03	0.238 ± 0.004
	2:1:1	72.87 ± 1.50	0.150 ± 0.028
	2:1:2	114.63 ± 0.11	0.367 ± 0.010
	2:1:5	120.91 ± 1.73	0.370 ± 0.016

The optical spectra of AB-LNPs and free BDP are shown in Figure 1b. AB-LNPs showed no absorption peak in H_2_O in the wavelength range of 500–800 nm. However, a typical absorption peak of BDP appeared when AB-LNPs were dissolved in DMSO, demonstrating that BDP was successfully grafted in AB-LNPs. To our surprise, the typical absorption peak of Au nanoclusters at about 550–650 nm disappeared in AB-LNPs in H_2_O. The binding of hydrophobic BDP onto Au-LNPs might affect the light absorption of Au nanoclusters. The loading efficiency of BDP in AB-LNPs determined by using UV–vis measurements (λ_ex_ = 600 nm) is 51 ± 1.2% (*n* = 3). A TEM image of AB-LNPs is shown in Figure 1c. Particles with diameters of ca. 10 nm were observed for AB-LNPs. AB-LNPs showed a rice-like shape, which was similar to that of LNPs and Au-LNPs, indicating that grafting both Au^3+^ and BDP onto LNPs did not destroy the structure of the preformed LNPs.

### Photothermal properties of AB-LNPs

**Figure 2:**
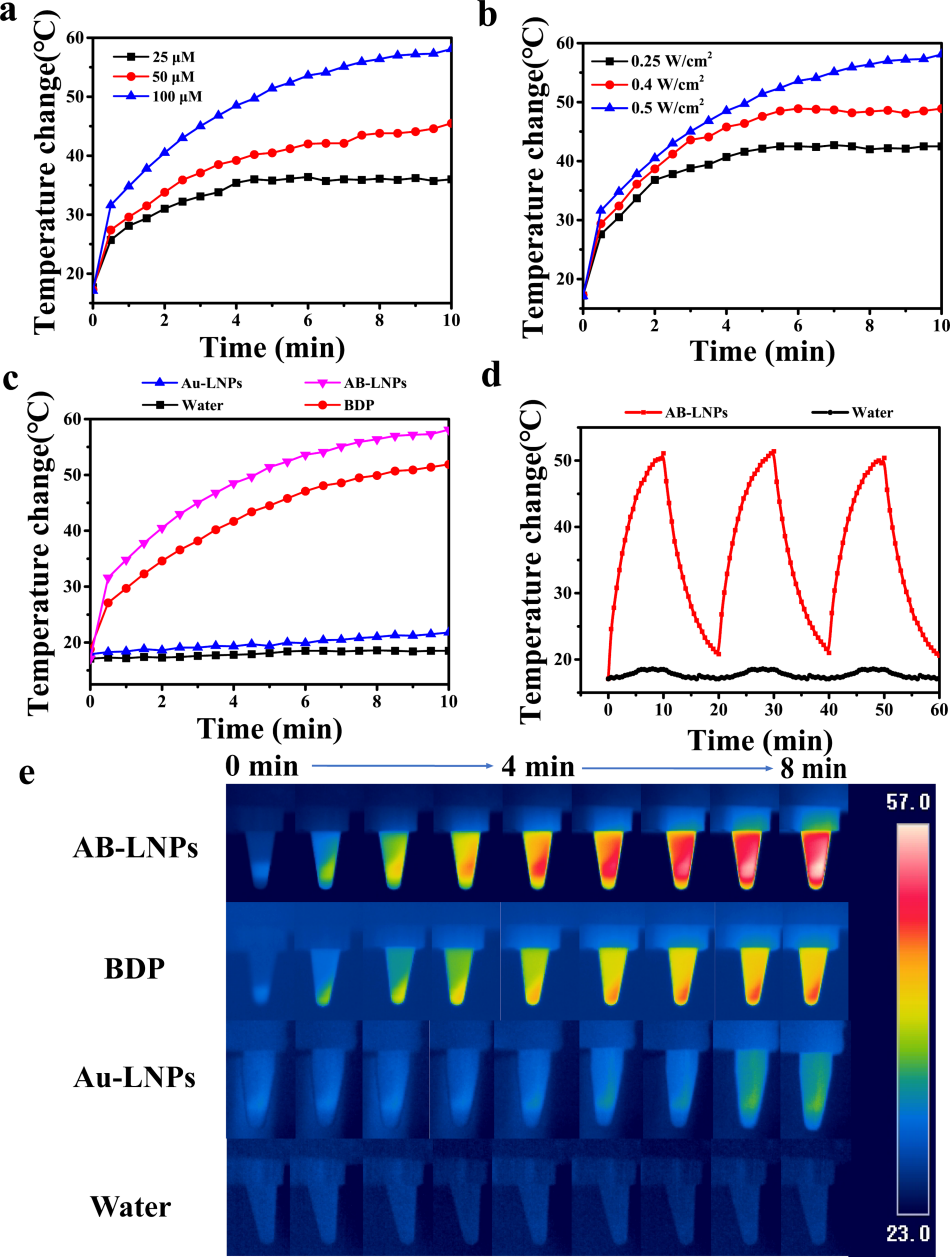
Photothermal properties of AB-LNPs. (a) Photothermal heating curves for AB-LNPs at different BDP concentrations with laser irradiation for 0–10 min (680 nm, 0.5 W/cm^2^). (b) Photothermal heating curves for AB-LNPs under 680 nm laser irradiation at different power densities for 0–10 min. (c) Photothermal heating curves of water, Au-LNPs (100 μM), BDP (100 μM), and AB-LNPs after laser irradiation (680 nm, 0.5 W/cm^2^). (d) Photothermal stability of AB-LNPs under laser irradiation (680 nm, 0.5 W/cm^2^) for three alternating ON/OFF cycles. (e) Thermal images of water, Au-LNPs (100 μM), BDP (100 μM), and AB-LNPs under laser irradiation (680 nm, 0.5 W/cm^2^). AB-LNPs in (b–e) contain 100 μM Au and 100 μM BDP.

To evaluate the photothermal properties of AB-LNPs, the temperature changes of each sample were recorded at various BDP concentrations (25, 50, and 100 μM) under the same laser irradiation (680 nm, 0.5 W/cm^2^). As illustrated in Figure 2a, it was observed that with an increase of AB-LNPs concentration, the temperatures increased greatly. After 10 min of laser irradiation (0.5 W/cm^2^), the temperature of the AB-LNP water suspension (with a BDP concentration of 100 μM and a Au concentration of 100 μM) showed an increase of around 58 °C, suggesting a good photothermal effect of AB-LNPs. Moreover, the temperature changes of AB-LNPs at a BDP concentration of 100 μM under different power intensities (0.25, 0.4, and 0.5 W/cm^2^) were measured (Figure 2b). It is evident that the temperature of the AB-LNP suspension increases quickly with the increase of light intensity.

For comparison, the photothermal effects of Au-LNPs (100 μM Au) and free BDP (100 μM) under 680 nm laser irradiation (0.5 W/cm^2^) were also evaluated. The temperature of Au-LNPs reached 23 °C and the temperature of free BDP samples reached 48 °C after 10 min irradiation (Figure 2c). In contrast, water showed only a slight temperature increase of 1–2 °C. The results demonstrated that AB-LNPs showed enhanced photothermal effects compared to free BDP and Au-LNPs. We further evaluated the photothermal stability of AB-LNPs under laser irradiation (680 nm, 0.5 W/cm^2^) for three alternating ON/OFF cycles (Figure 2d). The temperature changes of AB-LNPs (containing 100 μM BDP and 100 μM Au) were consistent in each cycle, indicating the excellent photothermal stability of AB-LNPs. Thermal images of water, BDP, Au-LNPs, and AB-LNPs under laser irradiation (680 nm, 0.5 W/cm^2^) were shown in Figure 2e, which also demonstrated the best photothermal effects of AB-LNPs. The results proved that the combination of Au and BDP in one LNP platform could yield synergized photothermal effects.

### Stability of AB-LNPs

**Figure 3:**
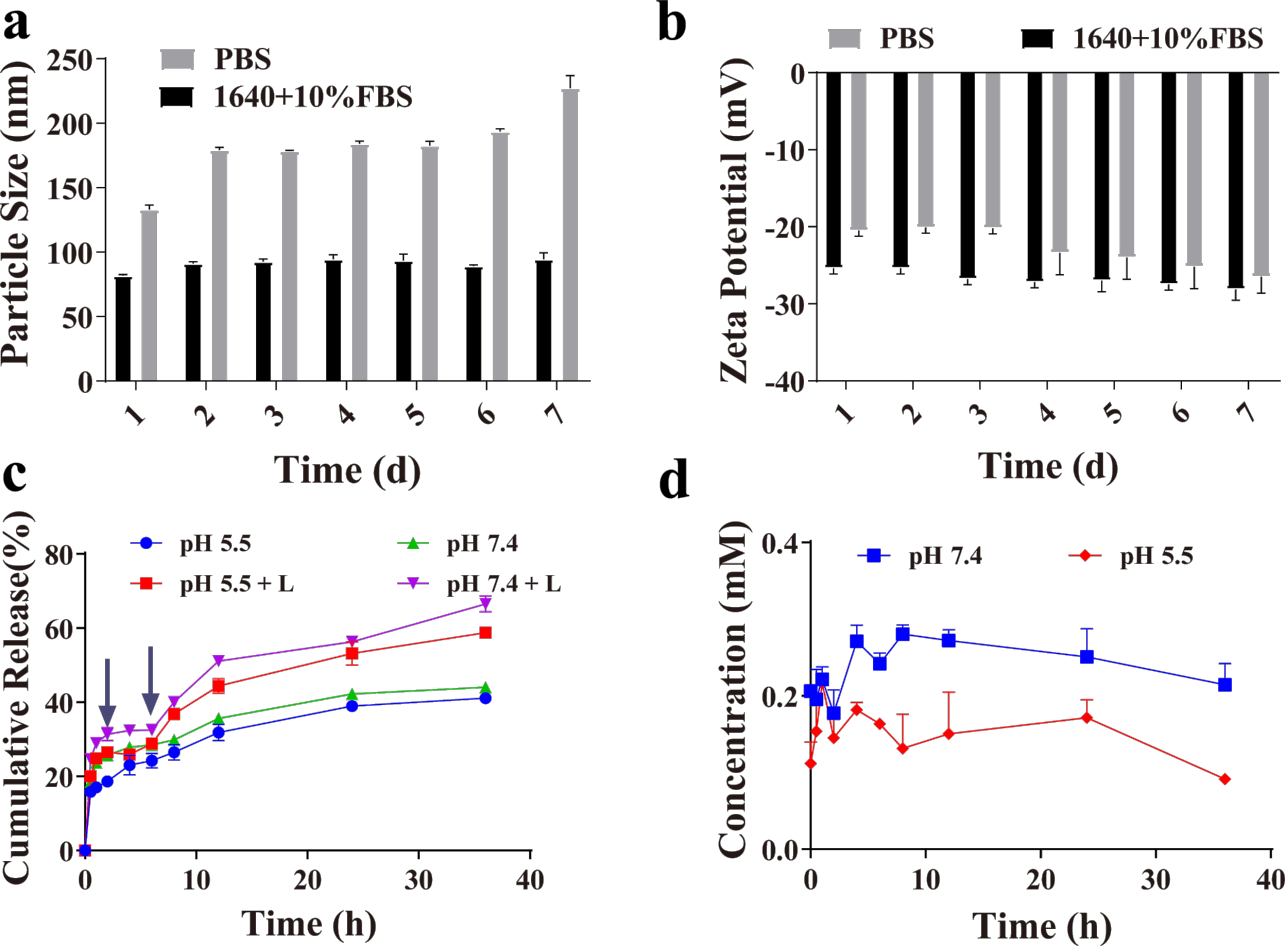
Stability and drug release properties of AB-LNPs. Changes of (a) particle size and (b) zeta potential of AB-LNPs in PBS or RPMI-1640 medium supplemented with 10% FBS over a period of 7 days. (c) Drug release profiles of BDP from AB-LNPs in PBS with pH 5.5 and 7.4 with or without exposure to 680 nm laser irradiation (0.5 W/cm^2^, 1 min) at 2 and 6 h. (d) Stability of free BDP in PBS with different pH.

The stability of AB-LNPs was evaluated by monitoring the particle size of AB-LNPs in two different media for 7 days. When AB-LNPs was dispersed in RPMI-1640 + 10% FBS for 7 days, there were no significant change in particle size. The particle size of AB-LNPs was still less than 100 nm after 7 days, indicating that AB-LNPs are stable under physiological conditions (Figure 3a). But the particle size increased obviously when AB-LNPs were dispersed in PBS. The salt ions may affect the interaction between Au-LNPs and BDP. No obvious change of the zeta potentials of AB-LNPs was found in two different media after 7 days (Figure 3b), suggesting that AB-LNPs maintained the nanoparticle structure in PBS and RPMI-1640 + 10% FBS for 7 days.

### Drug release properties of AB-LNPs

To investigate whether AB-LNPs could respond to laser irradiation and release the drug from LNPs, in vitro release studies of AB-LNPs were performed in PBS (pH 7.4 or 5.4) at 37 °C with or without 680 nm laser irradiation (0.5 W/cm^2^, 1 min). Two laser irradiations were carried out at 2 and 6 h for easier observation of the accelerated release. The laser irradiation accelerated the release of BDP from AB-LNPs. The accumulated released amount of AB-LNPs in pH 7.4 with laser irradiation was almost 65.7% at 36 h while the accumulated released amount of AB-LNPs without laser irradiation was only 50% (Figure 3c). The results can be attributed to the increased solution temperature, which accelerated the dissociation of BDP from AB-LNPs. There are no obvious differences between the release profiles of AB-LNPs at pH 5.5 and at pH 7.4, indicating that AB-LNPs do not exhibit pH-responsive drug release. The stimulus-responsive release characteristics could reduce the systemic toxicity of AB-LNPs on normal tissues. To check whether free BDP is stable during the release experiment, the content of BDP in PBS (pH 7.4 or 5.4) was measured using UV–vis spectroscopy. The content of free BDP showed little change over a period of 36 h (Figure 3d), indicating that BDP has good stability under these release conditions.

### Cellular uptake of AB-LNPs

**Figure 4:**
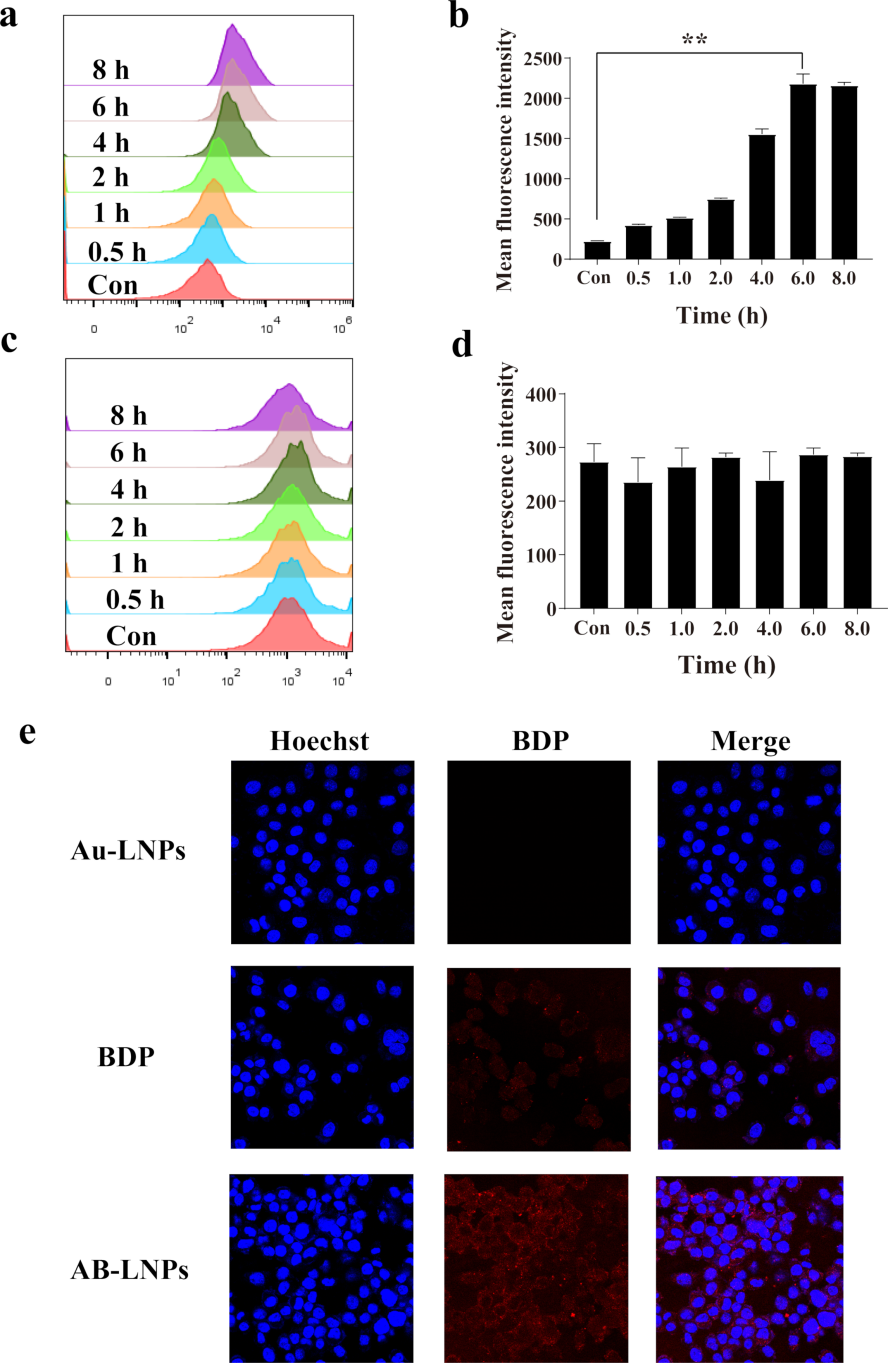
Cellular uptake of AB-LNPs. Cellular uptake efficiency of 4T1 cells treated with (a) AB-LNPs or (c) free BDP with different incubation times (0.5, 1, 2, 4, 6, and 8 h). Mean fluorescence intensity of 4T1 cells treated with (b) AB-LNPs or (d) free BDP for 0.5, 1, 2, 4, 6, and 8 h. (e) CLSM images of 4T1 cells after incubation with Au-LNPs, BDP, or AB-LNPs for 6 h. ** *p* < 0.01 compared with control by Student’s *t*-test.

Efficient cellular internalization of drugs is critical to the anticancer therapeutic effects [26–27]. From the above studies, we found that DSPE-DTPA/Au^3+^/BDP at a molar ratio of 2:1:1 could form uniform complex nanoparticles with suitable particle size (73 nm) for in vivo application. Besides, at this molar ratio, BDP could be successfully grafted onto the LNPs. Flow cytometry was employed to study the cellular uptake efficiencies of AB-LNPs and free BDP by detecting the fluorescence of BDP in the PerCP-Cy5-5 channel. 4T1 cells treated with AB-LNPs showed an evident increase in mean fluorescence intensity with the extension of the incubation time and reached the peak at 6 h (Figure 4a,b). The uptake amount of AB-LNPs after 6 h incubation time was nine times higher than that of the control, indicating the successful endocytosis of AB-LNPs into 4T1 cells. However, free BDP did not show time-dependent cellular uptake (Figure 4c,d). Compared with free BDP, AB-LNPs showed significantly enhanced uptake efficiency, indicating that the nanoparticles can increase the internalization and accumulation of drugs inside the cancer cells. The internalization of BDP or AB-LNPs in 4T1 cells was also observed by CLSM. The intracellular fluorescence signals of 4T1 cells treated with AB-LNPs were brighter than that of free BDP, indicating the enhanced internalization of BDP through entrapping in lipid nanoparticles, which was consistent with the results obtained from flow cytometry. For comparison, no fluorescence could be found in cells treated with Au-LNPs. AB-LNPs showed the highest cellular uptake efficiency after 6 h of incubation. Hence, we chose 6 h incubation time to study the anti-proliferative activity of AB-LNPs.

### Anti-proliferative activity of AB-LNPs

**Figure 5:**
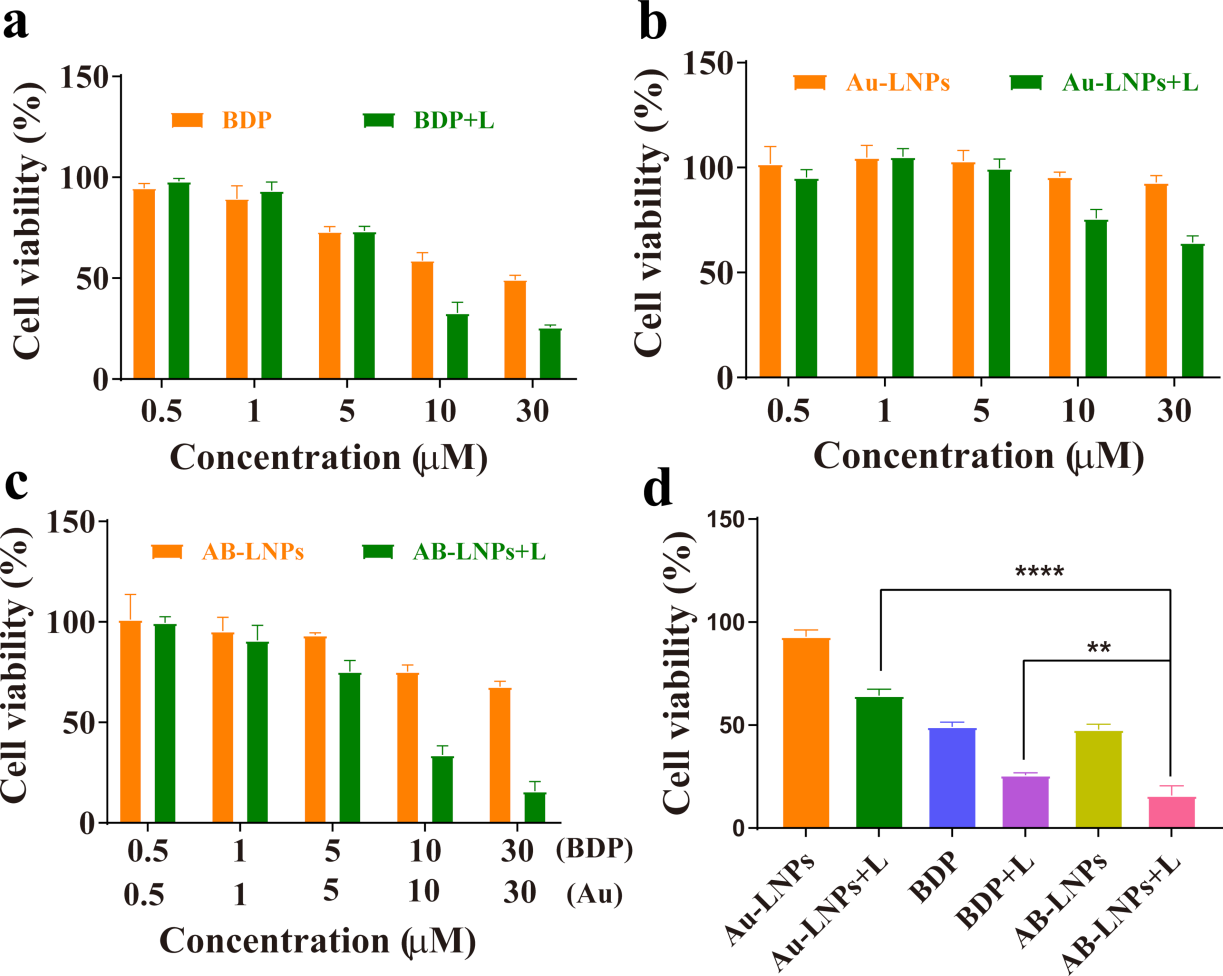
In vitro anti-proliferative activities of AB-LNPs with and without laser irradiation. (a) Cell viability of 4T1 cells after the treatment with BDP at concentrations of 0.5, 1, 5, 10, and 30 μM. (b) Cell viability of 4T1 cells treated with Au-LNPs at different concentrations of Au. (c) Cell viability of 4T1 cells treated with different concentrations of AB-LNPs. (d) Cell viability of 4T1 cells treated with BDP (30 μM), Au-LNPs (30 μM) and AB-LNPs (containing 30 μM BDP and 30 μM Au). Cells were cultured in dark environment with or without laser irradiation (680 nm, 0.5 W/cm^2^, 60 s). ** *p* < 0.01, **** *p* < 0.0001 compared with control by Student’s *t*-test.

MTT assay was employed to investigate the viability of 4T1 cells treated with BDP, Au-LNPs, and AB-LNPs for 24 h with or without laser irradiation (680 nm, 0.5 W/cm^2^, 60 s). A laser wavelength of 680 nm was used for PTT in this study. BDP showed concentration-dependent killing effects in cells with laser irradiation and caused 72.7% of cell death at the concentration equivalent to 10 μM (Figure 5a). At the highest concentration (30 μM of BDP), the cell viabilities of BDP with laser irradiation remained at about 25.75% after 24 h incubation under 680 nm laser irradiation. Au-LNPs exhibited almost no distinct cytotoxicity under all concentrations in the absence of light. Under laser irradiation, only a slight decrease in the cell viability could be found in Au-LNP-treated cells, indicating the limited phototoxicity of Au-LNPs (Figure 5b). In darkness, similar to BDP, AB-LNPs demonstrated slight dose-dependent toxicity to 4T1 cells. No obvious toxicity was found in AB-LNPs when the concentrations of BDP and Au were 10 μM and below. Under laser irradiation, the viability of cells treated with AB-LNPs decreased significantly in comparison to BDP or Au-LNPs at the same concentration, indicating the synergistic effects of BDP and Au-LNPs (Figure 5c). Because of the photothermal effects of BDP and Au-LNPs, AB-LNPs with a BDP concentration of 30 μM and a Au concentration of 30 μM presented strong cell proliferation inhibitory effects (90.2%) in comparison to 30 μM of BDP and 30 μM of Au-LNPs (Figure 5d). The results obviously demonstrated that AB-LNPs under laser irradiation could trigger the photothermal activity of BDP and Au-LNPs to exert synergized PTT. To prove the synergistic effect of combining the BDP and Au-LNPs, we also calculated the combination index (CI) through calculating the IC_50_ (half maximal inhibitory concentration) values of BDP, Au-LNPs, and AB-LNPs under laser irradiation after 24 h of treatment. The CI was less than 1, indicating the synergistic effects of BDP and Au in AB-LNPs.

## Discussion

In this work, LNPs were used as a platform to deliver both AuNPs and BODIPY dye for synergized PTT. To our knowledge, this is the first report about using LNPs as carriers for the co-delivery of gold nanoclusters and BODIPY, the first investigation of the synergistic thermal effects of gold nanoclusters and BODIPY, and the first development of Au- and BODIPY-grafted LNPs using a very simple and convenient method.

PTT-induced direct anti-tumor effects through photothermal ablation or hyperthermia have been widely studied [28]. AuNPs are one of the most attractive PTAs for biomedical applications because of their biocompatibility and distinctive optical properties. However, AuNPs are easily aggregate and are difficult to eliminate in vivo. In a previous reported work, we synthesized LNPs grafted with tiny gold nanoclusters (Au-LNPs) to solve issues regarding aggregation and metabolism [8]. However, PTT of Au-LNPs is limited by the low photothermal temperature, which usually requires high doses or prolonged illumination time. BODIPYs have raised great interest in cancer phototherapy because of unique physiochemical characteristics, excellent photothermal converstion ability, and easy chemical modification. To improve the water solubility of BODIPYs and increase their tumor targeting ability, lipid nanoparticulate delivery systems have been reported to encapsulate BODIPYs for in vivo use [13]. Therefore, we chose BODIPY as a synergic agent to obtain synergistic PTT. Au-LNPs were mixed with BODIPY to form stable AB-LNPs. AB-LNPs showed high cellular uptake efficiency (Figure 4) and good synergistic PTT (Figure 5).

BODIPY compounds possess diverse structures, adjustable spectra, and excellent photostability. Therefore, researchers have been committed to improving the photothermal performance of BODIPY for effective PTT [29]. Two strategies including fabricating BODIPY with halogenated substitutions and modification of BODIPY with heavy metal atoms have been employed to decrease the radiative transitions and enhance the phototherapeutic effects. Halogenated BODIPY ensures efficient singlet-to-triplet intersystem crossing or non-radiative transitions for better photothermal conversion efficiency [13–14]. In this work, we chose a halogenated BODIPY with good photothermal conversion rate to be grafted onto Au-LNPs for synergistic PTT.

In current study, we attempted to create AB-LNPs by simply grafting Au and BDP onto the surface of LNPs. The particle size depended on the molar ratio of DSPE-DTPA/Au^3+^/BDP (Table 1). The particle sizes of AB-LNPs changed with the amount of the grafted BDP on the surface of Au-LNPs. AB-LNPs with optimal molar ratio showed appropriate particle size, monodisperse distribution, rice-like shape, and high drug loading efficiency (Figure 1). AB-LNPs also showed good stability under physiological conditions and demonstrated laser-responsive release characteristics (Figure 3). BDP grafted on the AB-LNPs could generate greatly enhanced cellular uptake in cancer cells (Figure 4). Due to the enhanced permeability and retention effect (EPR) of tumors, AB-LNPs will have a good tumor accumulation ability. The enhanced cellular uptake efficiency and the laser-responsive release properties of AB-LNPs could further improve the anti-tumor effects.

The clinically applicable temperatures for hyperthermia range from 43 to 47 °C since heating above 43 °C could induce apoptosis in cancer cells [30]. AB-LNPs showed excellent photothermal effects and photothermal stability with a temperature increase beyond 58 °C (Figure 2). Both Au- and BDP-grafted on LNPs could absorb light and convert the light energy into heat to achieve synergized PTT (Figure 5). The enhanced cellular uptake of AB-LNPs by cancer cells (Figure 4) and a high concentration of BDP being released into the cells after laser irradiation (Figure 3) also contributed to the improved photothermal therapeutic effects of AB-LNPs. Furthermore, heat produced by laser irradiation could increase membrane fluidity, which could, in turn, promote the cellular uptake efficiency. The good photothermal effects and photothermal stability, the light response release properties, and the efficient cellular uptake of AB-LNPs are all beneficial to the synergistic PTT effects.

Regarding the complex pathogenic mechanisms of cancer, synergistic PTT with two or more PTAs that suppress tumor cells via different pathways is an appealing strategy to treat cancer. Yu et al. coated prussian blue (PB) on NaNdF_4_ nanoparticles to fabricate core–shell nanocomplexes with improved photothermal conversion efficiency by generating new cross-relaxation pathways between the ladder-like energy levels of Nd^3+^ ions and the continuous energy band of PB [10]. The strategy to generate new cross-relaxation pathways between different materials is applicable to design all kinds of enhanced photothermal agents. In this work, AB-LNPs showed excellent photothermal therapeutic effects compared to monotherapy using only free BDP or Au-LNPs (Figure 5), demonstrating good synergistic PTT by combining Au-LNPs and BDP. The synergized PTT could reduce the dosage of AB-LNPs and decrease the toxicity of carrier materials. In addition, AB-LNPs could cause severe cytotoxicity when exposed to only a very low dose of laser irradiation (0.5 W/cm^2^, 60 s), which is beneficial to clinical application.

## Conclusion

AB-LNPs were prepared by simply mixing Au-LNPs with BDP. The physicochemical properties of AB-LNPs were closely related with the DSPE-DTPA/Au^3+^/BDP molar ratio. Using the optimal DSPE-DTPA/Au^3+^/BDP molar ratio of 2:1:1, AB-LNPs with appropriate particle size and monodisperse distribution could be prepared reproducibly at 25 °C. AB-LNPs demonstrated excellent stability, good photothermal conversion efficiency, and laser-responsive release properties. In 4T1 cancer cells, AB-LNPs showed enhanced cellular uptake efficiency in comparison to BDP and synergistic photothermal effects. The simple and adaptive nanoparticle design may have great potential for the treatment of cancer and other diseases.
